# Salivary Metabolomic Signatures Associated with Sex-Specific Psychological Distress in Syrian Refugees: A Proof-of-Principle Study

**DOI:** 10.3390/metabo16040216

**Published:** 2026-03-25

**Authors:** Tanzi D. Hoover, Steel M. McDonald, Laisa Kelly, Yesim Erim, Tony Montina, Gerlinde A. S. Metz

**Affiliations:** 1 Canadian Centre for Behavioural Neuroscience, Department of Neuroscience, University of Lethbridge, Lethbridge, AB T1K 3M4, Canada; hoovtd@uleth.ca (T.D.H.); steel1@ualberta.ca (S.M.M.); laisa.kelly@uleth.ca (L.K.); 2 Department of Chemistry and Biochemistry, University of Lethbridge, Lethbridge, AB T1K 3M4, Canada; 3 Department of Psychosomatic Medicine and Psychotherapy, Friedrich-Alexander University Erlangen-Nuremberg, 91054 Erlangen, Germany; yesim.erim@uk-erlangen.de; 4 Southern Alberta Genome Sciences Centre, University of Lethbridge, Lethbridge, AB T1K 3M4, Canada

**Keywords:** depression, anxiety, saliva, proton nuclear magnetic resonance (^1^H NMR), biomarkers, trauma, stress resilience, risk prediction

## Abstract

Background: Refugees arriving from conflict zones often continue to experience trauma and are at increased risk of anxiety and depression. Those seeking asylum form a group at higher risk of suffering adverse mental health outcomes, with higher needs for psychosocial and therapeutic care. This study aimed to determine metabolic changes potentially associated with psychological distress in refugees from Syria, using a saliva-based metabolomics approach via proton nuclear magnetic resonance (1H NMR) spectroscopy. Methods: Participants were recruited from Lethbridge Family Services and categorized into high and low stress burden groups using questionnaires assessing depression (PHQ-9) and generalized anxiety (GAD-7). Salivary metabolomic profiles from 26 female and 32 male participants were analyzed using supervised and unsupervised multivariate statistical methods to identify metabolic differences linked to composite stress, depression, and anxiety. Results: Salivary metabolic profiles showed the most prominent differences associated with anxiety in female participants and depression in male participants. Multivariate statistical analyses identified 31 metabolites and 13 biological pathways that were significantly altered according to mental health status, with the greatest changes observed in glycolysis/gluconeogenesis, sphingolipid metabolism, and taurine/hypotaurine metabolism. Conclusions: These findings indicate that salivary 1H NMR metabolomic profiling can identify a quantifiable “metabolic fingerprint” related to impaired mental health and psychological distress in a cost-effective, objective, and non-invasive way. This analytical strategy shows potential as a screening tool to support effective decision-making, enabling early identification of individuals at highest risk who require timely emotional and medical support.

## 1. Introduction

The Syrian conflict, which began in 2011, has displaced more than six million people and exposed refugees to prolonged instability, uncertainty, and loss of autonomy [1]. Such experiences markedly increase vulnerability to psychological distress, with elevated rates of anxiety, depression, and trauma-related symptoms consistently reported in resettled Syrian populations [2,3,4]. Following the arrival of approximately 60,000 Syrian refugees in Canada since 2015 [5], there is an increasing need for accessible, culturally appropriate approaches to mental health assessment.

Across multiple host countries, studies have shown substantially higher prevalence rates of depression, anxiety, and post-traumatic stress disorder (PTSD) among Syrian refugees compared to the general population [3,6,7]. Similar trends have been observed in other trauma-exposed groups, where psychological distress frequently co-occurs with increased rates of cardiovascular, respiratory, gastrointestinal, and other somatic health conditions [8,9,10,11]. Regardless of whether physical illness precedes or follows mental health challenges, their interaction underscores a broader pattern of heightened health vulnerability in displaced populations. Despite this burden, mental health diagnosis continues to rely primarily on clinical interviews and self-report instruments, such as the Patient Health Questionnaire (PHQ-9) for depressive symptoms [12,13,14] and the Generalized Anxiety Disorder 7-item scale (GAD-7) for generalized anxiety [15,16]. These tools are widely used and well validated, yet they share a core limitation: they depend on subjective reporting and do not capture the underlying biological states that may accompany psychological distress [17]. This reliance on symptom-based assessment presents challenges in culturally diverse or trauma-affected populations, where experiences of distress may be expressed or interpreted differently.

To address these gaps, growing interest focuses on integrating objective, biology-informed measures into mental health research. Metabolomics offers one such opportunity by identifying small-molecule signatures that reflect physiological responses to stress, inflammation, and overall health status [18,19,20,21,22,23]. Metabolomics has been successful in disease diagnosis, including neurological and psychiatric disorders, cancers, and metabolic and cardiovascular diseases [24,25,26,27,28]. When used alongside established psychometric instruments, metabolic biomarkers may enhance the precision of mental health assessment and support the development of more personalized, biologically informed care strategies [21,27,29,30]. The use of proton nuclear magnetic resonance (^1^H NMR) spectroscopy in metabolomics is particularly effective in untargeted discovery of biological signalling pathways or metabolic fingerprints linked to pathophysiological states using clinically accessible biofluids [31,32,33,34,35,36]. As saliva collection is the most accessible and least invasive clinical sample, it is becoming a prominent choice for large-scale, longitudinal biomarker identification [37,38,39]. Notably, saliva contains 308 quantifiable metabolites that change significantly in response to a variety of different physiological states and stressors, of which 76 can be detected and quantified by NMR analyses [40].

The present proof-of-principle study was designed to determine whether ^1^H NMR spectroscopy of saliva can be used to identify metabolomic changes associated with mental health outcomes among individuals from the Syrian refugee community. Participants were invited to complete psychometric assessments measuring symptoms of depression and generalized anxiety disorder, which were evaluated both individually and through a customized composite distress score. Based on these assessments, participants were grouped according to relative levels of psychological distress to identify salivary metabolites that may reflect symptoms of depression and anxiety. This approach seeks to integrate objective biological measures with lived experience to better understand and support the psychological well-being of individuals affected by forced displacement and trauma.

## 2. Materials and Methods

### 2.1. Participant Recruitment and Psychometric Assessments

This study was approved by the University of Lethbridge Human Participant Research Committee on 7 July 2021 (Protocol# 2019-065) and all participants provided informed consent. The study included 88 Syrian adults (female *n* = 44; male *n* = 44) recruited through Lethbridge Family Services—Immigrant Services. Each participant was invited to complete the PHQ-9 questionnaire as a validated screening instrument for depression and the GAD-7 questionnaire as a validated screening instrument for generalized anxiety disorder. These questionnaires were available to participants in English or Arabic. The session was facilitated with the support of translators fluent in Arabic and familiar with the Syrian cultural context of the participants. All participants were in good general health and reported no physical illness or disease such as diabetes, cardiovascular conditions, or infections.

Each assessment was scored based on the respective scoring guides, which was then used to help divide the participants into high- and low-symptomatology comparison groups for depression and anxiety and overall mental distress (a composite score). The PHQ-9 and the GAD-7 were each given a score from 0 to 3 with respect to symptom severity (0 being no prevalence, 1 being low, 2 being moderate, and 3 being severe). Composite distress scores were formulated based on a sum of both assessments, ranging from a score of 0–3 as low distress and 4–6 as high distress. Depression and anxiety comparisons were also considered independently based on their corresponding test. Low depression and anxiety were scored as 0 to 1, while high depression and anxiety were scored as 2 to 3.

### 2.2. Saliva Sample Collection

Saliva sample collection via Salimetrics SalivaBio Oral Swab kits (Salimetrics, State College, PA, USA) was performed [41] in the evening at the time when the psychometric assessments were completed. Because several salivary metabolites show diurnal variation, all samples were collected within a standardized evening time window to reduce circadian-related variation [42]. Participants were instructed to abstain from eating, drinking, smoking, chewing gum, or engaging in oral hygiene activities for at least one hour prior to saliva sample collection. Prior to sample collection, each participant rinsed their mouth with water and waited approximately ten minutes to ensure proper salivary flow. Salivettes were then given and placed under participants’ tongues. After two minutes, the salivettes were placed in conical tubes and kept on dry ice for transport and were subsequently stored at −80 °C until further processing.

### 2.3. Sample Preparation

Samples were removed from a −80 °C freezer and allowed to thaw to room temperature. To collect the saliva, salivettes were centrifuged for 15 min at 1500× *g*. For each sample, 200 μL of collected saliva and 300 μL of phosphate buffer were pipetted into a 0.5 mL 3 kDa centrifuge filter and centrifuged at 14,000× *g* for 30 min at 4 °C. All centrifuge filters were washed 10 times with deionized water immediately to remove the glycerol preservative from the filters. 380 μL of the filtrate, 100 μL of additional buffer, and 120 μL of D_2_O were pipetted into 2 mL microfuge tubes and centrifuged at 14,000 rpm for 15 min at 4 °C. Phosphate buffer was prepared as a 4:1 ratio of KH_2_PO_4_:K_2_HPO_4_ in a 4:1 H_2_O:D_2_O solution to obtain a final concentration of 0.5 M. The D_2_O contained a weight per volume (*w*/*v*) ratio of 0.02709 trimethylsilylpropanoic acid (TSP) as a chemical shift reference for ^1^H NMR spectroscopy (Cambridge Isotopes Laboratories Inc., Tewksbury, MA, USA). Sodium azide (NaN_3_, 0.03% *w*/*v*) was added as an antimicrobial agent, and total buffer pH was titrated to 7.40 using 3 M HCl. Following centrifugation, the prepared samples were returned to the −80 °C freezer for future ^1^H NMR analysis. Of the total samples collected (*n* = 88), 30 samples (18 female, 12 male) had an insufficient amount of saliva extracted from the swab for NMR analysis (new *n* = 58; female *n* = 26, male *n* = 32). On the day of sample analysis in the ^1^H NMR spectrometer, the microfuge tubes were removed from the −80 °C freezer, allowed to thaw at room temperature and 550 μL of supernatant was transferred to labelled 5 mm NMR tubes.

### 2.4. NMR Data Acquisition and Processing

The data were acquired using a 700 MHz Bruker Avance III HD spectrometer (Bruker BioSpin GmbH, Rheinstetten, Germany), equipped with a 5 mm triple resonance TBO-Z probe. The Bruker 1-D NOESY gradient water suppression pulse sequence ‘noesygppr1d’ was used. The data were collected according to the procedures outlined by Paxman et al. (2018) [22], except that the total number of scans was increased to 512. The spectra were phase-shifted, baseline-corrected, and line-broadened by 0.3 Hz using TopSpin 3.5 patch level 7 (Bruker BioSpin, Billerica, MA, USA). All spectra were converted to ascii files and exported to MATLAB R2015b (MathWorks, Natick, MA, USA) for further analysis. The spectra underwent dynamic adaptive binning (DAB) [43], followed by manual adjustment to correct for any errors in the algorithm. This process utilized spectral features and an objective function in an unbiased and untargeted way to create bins that contain as few metabolite peaks as possible. The bin containing the water peak was removed, resulting in a total of 332 bins for all saliva spectra. The spectra were normalized to the total area of all bins, log-transformed, and Pareto-scaled. All spectra peaks were referenced to TSP (0.00δ) [44].

### 2.5. Statistical Analysis

Three hundred thirty-two bins in NMR spectra were first analyzed for all comparison groups and deemed significant or non-significant using a decision tree algorithm [45]. The 332 bins were tested for normality using the Shapiro–Wilk test, followed by the Mann–Whitney U test. All *p*-values were Bonferroni–Holm-corrected for multiple comparisons with the threshold for significance at 0.05. As an additional bin selection method, variable importance analysis based on random variable combination (VIAVC) was used [46]. The framework of VIAVC includes binary matrix resampling, which (1) guarantees that each variable has been selected with the same probability and generated different variable combinations, (2) the importance of each variable by assessing the area under the curve (AUC) of the receiver operating characteristic (ROC) curve [47], and (3) produces a ranking of the final remaining informative variables. The VIAVC algorithm was carried out using Pareto scaling, double ten-fold cross validation, and binary matrix resampling set to 1000. The VIAVC algorithm first carries out 10-fold cross-validation to create smaller set of variables based on the modelling of the 1000 randomly created subsets. Variables are removed if they are determined to be uninformative or interfering in the model (they lead to reduced classification of the groups). Once this reduced subset of variables is determined, VIAVC then applies 10-fold double cross-validation. This application of double ten-fold cross validation ensures that each predicted sample in the test set is not part of the calculation of the number of coefficients and components utilized in the model, which is determined by the training and validation set. VIAVC repeats this double cross-validation procedure until each sample has been included in the test set at least once. This final step of the VIAVC algorithm produces the best subset of variables that are a robust set of classifiers for the model. The *p*-value utilized as the threshold for statistical significance in the VIAVC testing was 0.05.

Model classification was determined first using unsupervised Principal Component Analysis (PCA), second using a supervised orthogonal projections to latent structures discriminant analysis (OPLS-DA) [48], and third using the area under ROC curves (AUC), all of which were generated in MetaboanalystR 3.0 [49]. Given the supervised nature of OPLS-DA and its tendencies to overfit the data, all OPLS-DA modelling underwent double ten-fold cross-validation testing to ensure the reliability of the model. The Human Metabolome Database and the Chenomx 8.5 NMR Suite (Chenomx Inc., Edmonton, AB, Canada) were used for metabolite identification [50,51]. Pathway topology analysis was completed using MetaboAnalyst by selecting the Kyoto Encyclopedia of Genes and Genomes (KEGG) database for *homo sapiens* as the library, the hypergeometric test for over-representation analysis, and relative betweenness for the topology analysis. The *p*-value utilized as the threshold for pathway topology analysis was 0.1.

## 3. Results

### 3.1. Subjects

Of the total samples collected (*n* = 88), 30 samples (18 female, 12 male) had an insufficient amount of saliva extracted from the swab for NMR analysis (new *n* = 58; female *n* = 26, male *n* = 32). Due to several incomplete surveys, only those who completed all sections of the PHQ-9 and GAD-7 questionnaires were considered for further analysis. The final composite comparison therefore included 21 females (11 low and 10 high scores) and 31 males (21 low and 10 high scores). The depression comparison included 26 females (15 low and 11 high levels) and 32 males (18 low and 14 high levels). Finally, the anxiety comparison included 26 females (16 low and 10 high levels) and 32 males (25 low and 7 high levels). Table 1 shows a summary of the sociodemographic variables deemed to possibly influence the metabolic results of participants in the study. This information was obtained at the time of sample collection. In some cases, not all participants provided complete sociodemographic information, and this is indicated in the table by a reduced total sample size (*n*) for the following: age, accommodation, and ethnicity.

### 3.2. Mental Health and Distress Have a Long-Term Impact on Metabolomic Profiles

**Figure 1:**
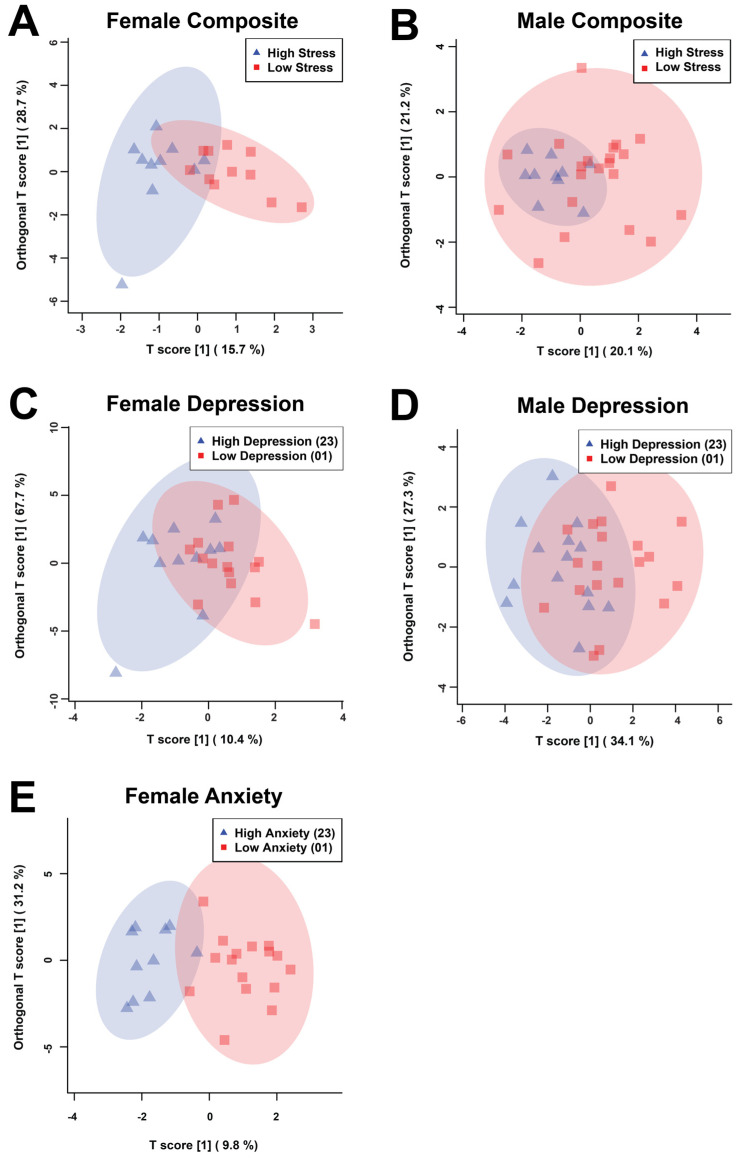
Supervised OPLS-DA score plots derived from the bins determined to be significant via Mann–Whitney U test (MW) or the Variable Importance Analysis based on random Variable Combination (VIAVC) for female (**A**,**C**,**E**) and male (**B**,**D**) salivary metabolite comparisons illustrating the variations due high and low composite distress test scores (**A**,**B**), depression scores based on the PHQ-9 (**C**,**D**), and anxiety scores based on the GAD-7 (**E**). Each triangle or square represents one individual. The percentages shown along the *x*- and *y*-axes indicate the amount of variance in the data set given by the between group and within group variation, respectively, and the shaded ellipses designate the 95% confidence interval of each group.

**Figure 2:**
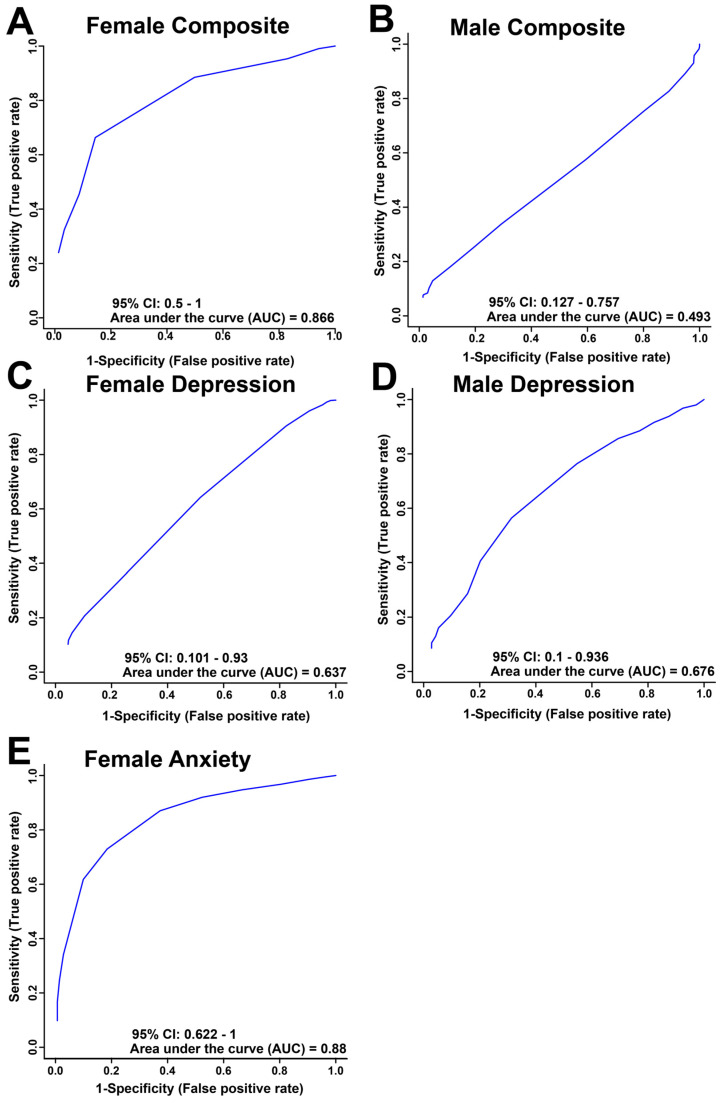
The receiver operator characteristics (ROC) curve derived from the metabolites determined to be significant by the VIAVC analysis for female (**A**,**C**,**E**) and male (**B**,**D**) salivary metabolite comparisons illustrating the sensitivity, specificity and predictive accuracy for high and low composite distress scores (**A**,**B**), depression scores based on the PHQ–9 (**C**,**D**), and anxiety scores based on the GAD–7 (**E**).

**Figure 3:**
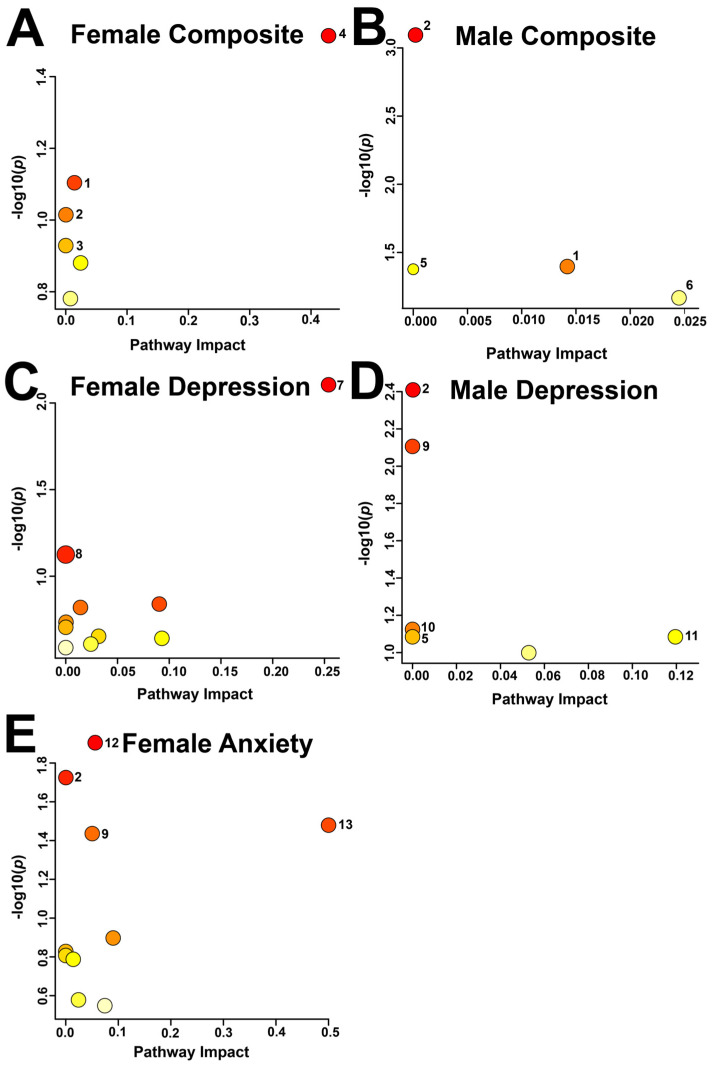
Pathway Topology Analysis of the identified metabolites deemed significant by MW or VIAVC testing for female (**A**,**C**,**E**) and male (**B**,**D**) salivary metabolite comparisons for high and low composite distress test scores (**A**,**B**), depression scores based on the PHQ-9 (**C**,**D**), and anxiety scores based on the GAD-7 (**E**). A higher value on the *y*-axis represents a lower *p*-value and the *x*-axis gives the pathway impact. A larger circle indicates a higher impact, and a deeper red circle indicates a lower *p*-value. Only metabolic pathways with *p* < 0.1 are labelled. The numbering scheme correlates with the following pathways: 1: Sphingolipid metabolism; 2: Glycolysis/Gluconeogenesis; 3: Glyoxylate and dicarboxylate metabolism; 4: Taurine and hypotaurine metabolism; 5: Pyruvate metabolism; 6: Glycerophospholipid metabolism; 7: Terpenoid backbone biosynthesis; 8: Caffeine metabolism; 9: Amino sugar and nucleotide sugar metabolism; 10: Fructose and mannose metabolism; 11: Pentose phosphate pathway; 12: Beta-Alanine metabolism; 13: Riboflavin metabolism.

The statistical analysis included 332 bins created from the spectra of the salivary metabolomic profiles. The bins determined to be significant by either the univariate Mann–Whitney U (MW) test or the multivariate variable importance analysis based on random variable combination (VIAVC) tests were used to construct orthogonal projections to latent structures discriminant analysis (OPLS-DA) scores plots for all comparisons (Figure 1, Supplementary Table S1). The male anxiety comparison group failed to pass permutation testing and was therefore not continued for analysis. These tests examined which bins led to differences in metabolomic profiles between the associated comparison groups. These analyses resulted in the following subdivision of bins: female composite (Figure 1A)—2 MW and 8 VIAVC bins; male composite (Figure 1B)—3 MW and 8 VIAVC bins; female depression (Figure 1C)—2 MW and 15 VIAVC bins; male depression (Figure 1D)—10 MW and 5 VIAVC bins; and female anxiety (Figure 1E)—7 MW and 22 VIAVC bins.

In male composite distress scores, there was complete overlap between the high and low groups, as indicated by both the poor quality of the model (Q^2^) and variation explained by the model (R2) that did not pass cross-validation (Q^2^ = −0.114, *p* = 0.286; R^2^ = 0.203, *p* = 0.433), whereas female composite distress scores had more notable group separation and passed cross-validation (Q^2^ = 0.388, *p* = 0.0035; R^2^ = 0.626, *p* = 0.007). For depression, male participants had more discernable separation than their composite distress scores and passed cross-validation (Q^2^ = 0.115, *p* = 0.0355; R^2^ = 0.366, *p* = 0.0435), and female participants still had separation that passed cross-validation (Q^2^ = 0.199, *p* = 0.0225; R^2^ = 0.396, *p* = 0.016), but the separation was less than that observed for their composite distress scores. Female anxiety was found to have the best group classification and passed cross-validation testing (Q^2^ = 0.254, *p* = 0.0115; R^2^ = 0.765, *p* = 0.0025). It should be noted that all of these Q2 values are relatively low and any group separation should be interpreted cautiously.

Receiver operator characteristic (ROC) curves were used to determine the sensitivity, specificity and accuracy of each comparison model using the metabolite bins identified as significantly altered by VIAVC testing (Figure 2). The comparisons of high and low for both female and male composite distress and depression and female anxiety revealed an area under the curve (AUC) and 95% confidence interval (shown in brackets) of 0.866 (0.5–1; Figure 2A), 0.493 (0.127–0.757, Figure 2B), 0.637 (0.101–0.93, Figure 2C), 0.676 (0.1–0.936, Figure 2D) and 0.88 (0.622–1, Figure 2E), respectively. The ROC curve for both the female distress composite and anxiety scores showed relatively good group classification with AUC values above 0.85, while depression in females and both male models showed poor classification with AUC values less than 0.7.

### 3.3. Pathway Analysis Reveals Sex Differences in the Metabolic Response to Distress

Metabolic pathway topology analysis identified 13 pathways that were potentially impacted across the different comparison groups (Figure 3, Table 2), based on an α-threshold of 0.1. In the female composite distress comparison, the potentially impacted pathways were taurine and hypotaurine metabolism, followed by sphingolipid metabolism and glycolysis/gluconeogenesis. In the male composite distress comparison, glycerophospholipid metabolism, sphingolipid metabolism, and glycolysis/gluconeogenesis were potentially altered.

In the female low vs. high anxiety comparison, the following four pathways were altered: glycolysis/gluconeogenesis; amino sugar and nucleotide sugar metabolism; beta-alanine metabolism; and riboflavin metabolism. Terpenoid backbone biosynthesis and caffeine metabolism were the only potentially impacted pathways in the female low vs. high depression comparison. The male low vs. high depression comparison indicated that glycolysis/gluconeogenesis and the pentose phosphate pathways were impacted. 

## 4. Discussion

By combining objective biological indicators with insights from lived experience, the present proof-of-principle study provides an expanded perspective on mental distress among individuals affected by trauma and forced displacement. The novelty of this study lies in the identification of sex-specific salivary biomarkers as indicators of mental well-being among Syrian refugees. The study adopted standardized questionnaires and a composite score as indicators of mental distress in Syrian refugees along with a metabolomics approach via ^1^H NMR spectroscopy. The present ROC models demonstrated differences in salivary metabolic profiles, with the most robust group classification observed for anxiety symptomatology and composite scores among female participants. In total, 63 out of 332 bins were labelled as containing significant metabolic differences through multivariate and univariate analysis. 31 unique metabolites were identified from the 63 bins during metabolite identification. These results suggest that salivary metabolic profiles may provide an objective supplementary method to aid in the diagnosis of depression and anxiety in particularly vulnerable populations. However, it is important to note that metabolic group separations reflect variation in self-reported outcomes rather than categorical diagnostic differences, and findings therefore represent exploratory patterns rather than definitive metabolic signatures of adverse mental health outcomes.

Many of the identified metabolites and their associated pathways are interrelated, which may be explained by the observation that depression and anxiety are often comorbid and share common risk factors, such as psychological distress [52,53,54]. In contrast, some of the identified pathways also enhance further understanding of the disparity between depression and anxiety. Although it is unlikely that the study of metabolites alone would be sufficient to predict the complex phenotype of distress vulnerability, including transition from at-risk states to clinically diagnosable mental disorders [27,55], the present results suggest specific metabolites that may contribute to the pathogenesis or symptomatology of depression and anxiety to guide future research.

### 4.1. Energy Metabolism and Mitochondrial Dysfunction

The present findings indicate that metabolites found in glycolysis and gluconeogenesis were the most frequently disrupted, expressing differences in the metabolomes between high and low distress across all comparisons. Glucose concentrations were lower in male participants with depression and higher in female participants with anxiety. Although the present study measured glucose in saliva rather than in the brain, disturbances in glycolysis/gluconeogenesis pathways have also been reported in psychiatric research examining systemic and central energy metabolism [56,57,58]. Salivary glucose reflects peripheral metabolic processes and cannot be interpreted as a direct marker of brain glucose utilization. However, the pathway-level alterations observed here may parallel broader metabolic dysregulation associated with depression and anxiety, as described in studies of systemic glucose regulation and cerebral energy demand [59]. These findings should therefore be viewed as preliminary, hypothesis-generating indicators of altered energy metabolism rather than evidence of direct changes in brain glucose levels. In contrast, higher availability of glucose in females may reflect an altered brain energy metabolism, as suggested by experimental findings in mice with elevated anxiety-like behaviours [59,60]. Chronically perturbed glucose concentrations may damage mitochondria and mitochondrial DNA, generating by-products that promote systemic inflammation, oxidative stress, and accelerate cellular ageing [8,61]. In cases of metabolic dysfunction or neuroinflammation, glucose may accumulate if utilization is impaired. A combination of altered glucose regulation and mitochondrial dysregulation may induce deficient energy utilization in times of higher energy demand, as previously observed in females with anxiety [60].

Components of mitochondrial dysfunction and inflammation may play a role in depression, anxiety, and other mental health conditions [8,62,63,64]. Brain tissue has a high energy demand for maintenance of transmembrane potentials, signal transduction, and synaptic plasticity. Gardner and Boles suggested several pathophysiological mechanisms underlying mitochondrial function in major depression [62]. Mitochondria play a pivotal role in cellular energy metabolism, neuronal amino acid, lipid, and steroid metabolism, modulation of cellular calcium levels, and the regulation of apoptosis [65,66,67]. It was suggested that impaired mitochondrial function might lead to disrupted neural plasticity and reduced cellular resilience, which may promote the development and progression of mood disorders [64]. The disturbance of the energy supply to the mitochondria, either as a cause or consequence of depression, may contribute to the pathophysiology of depressive symptoms [63].

Lactate is generally regarded as an indicator for the adaptive response of energy failure. Hence, the reduced lactate levels in male participants may reflect the energy supply/demand imbalance that potentially accompanies their depression [68,69]. In humans, aerobic glycolysis takes place in oxygen-rich cellular environments, whereas anaerobic glycolysis occurs during a lack of available oxygen, reducing pyruvate to lactate by the enzyme lactate dehydrogenase [70]. Glial cells, using the lactate shuttle, are responsible for transforming glucose into lactate and providing it to neurons. These observations support the finding of reduced glucose availability and corresponding decrease in lactate levels in males with depression.

Because saliva is a peripheral biofluid, metabolite variations observed here should not be interpreted as direct indices of brain metabolism. Salivary glucose [71,72] and lactate [73,74] show modest positive correlations with plasma levels in several physiological contexts, including glycemic monitoring and exercise-induced metabolic shifts. However, salivary concentrations can also be influenced by oral microbiome activity, salivary gland metabolism, mucosal permeability, nutritional status, and sympathetic activation. Similarly, phosphoethanolamine and taurine may reflect peripheral inflammatory processes, dietary factors, or oxidative stress rather than central nervous system activity. Thus, the present findings can be interpreted as exploratory indicators of peripheral metabolic differences associated with symptom severity rather than providing evidence of altered brain metabolite concentrations.

### 4.2. Sphingolipid and Glycerophospholipid Metabolism

Sphingolipids and glycerophospholipids are the most common lipids in brain membranes and have a larger role in neuronal activity, cellular signalling [75,76], signal transduction, cell growth, cell death [77], and disease pathologies [78,79]. O-phosphoethanolamine, which had altered concentrations in both the male and female composite comparisons, is an important precursor to the glycerophospholipid phosphatidylethanolamine. In the CDP-ethanolamine pathway, the enzyme ethanolamine-phosphate cytidylyltransferase converts phosphoethanolamine into CDP-ethanolamine, which is then subsequently converted to phosphatidylethanolamine by ethanolamine phosphotransferase [80,81]. Previous studies focusing on peripheral lipid metabolism in depressive patients have found an increase in activity compared to controls [53]. Levels of lipids and their associated enzymes can account for the severity of depression and measure the success of potential treatments [82]. Further work has shown a correlation between sphingolipids and inflammatory processes within the brain, involving either lipid cleavage or lipid turnover [77]. Moreover, clinically effective antidepressants inhibit sphingolipid production in the hippocampus, which may be essential for their therapeutic efficacy. Accordingly, membrane-forming lipids have high potential in the treatment of anxiety disorders [53]. Therefore, lipid-based therapies may offer personalized treatment, such as targeted dietary supplementation of n-3 polyunsaturated fatty acids [83], or pharmacological interference of lipids, such as glycerolipids, with lipid-regulating enzymes [78,84].

### 4.3. Taurine/Hypotaurine Metabolism

Taurine was highly upregulated in the female composite distress comparison, and taurine and hypotaurine metabolism emerged as the most significantly altered pathway, showing both the largest pathway impact and the lowest *p*-value. Taurine is an abundant central amino acid involved in synaptic inhibition and has been shown to increase significantly under stressful conditions [85], suggesting a critical role in neuroprotection [86]. It also acts as an inhibitory neuromodulator with known antianxiety properties [87] and may mitigate glutamate-induced mitochondrial damage and neuronal cell death by regulating intracellular calcium levels. In addition, taurine contributes to several key neurophysiological processes, including osmotic balance, membrane stabilization, detoxification, and neuromodulation [88]. These findings point to taurine as a potentially sex-specific neuroprotective metabolite that may buffer the effects of stress particularly in females.

### 4.4. Sex Differences

Sex differences usually are highly associated with a discriminatory metabolite signature in healthy humans and therefore should be considered in metabolomics studies using both male and female participants [89,90]. Overall, the current findings indicate that female participants had greater metabolic discrepancy in relation to mental distress severity compared to male participants, suggested by the statistical significance portrayed in Figure 1 and Figure 2. This does not indicate that female participants were more emotionally influenced by psychiatric distress than males; but rather, they generally showed greater variation than male participants in metabolites representing mental distress severity through a salivary ^1^H-NMR-based technique from a metabolic perspective, especially when investigating the discrepancy of composite distress and anxiety scores. Earlier investigations of sex differences in metabolites indicated that sex-specific patterns may be correlated to the effects of sex hormones, as sex differences emerge during reproductive years and gradually decline after menopause [91]. These findings underscore the importance of incorporating sex-specific analyses in metabolomic research on mental health, particularly in marginalized and vulnerable populations, as they reveal underlying biological variation that may inform more precise, personalized approaches to diagnosing and managing psychiatric distress and trauma.

### 4.5. Limitations

The present proof-of-principle study offers novel insights into the mechanistic pathways underlying mental health and vulnerability to distress. Nevertheless, the relatively small sample size highlights the need for further investigation before any clinical applications can be considered. While psychometric questionnaires are commonly used to assess mental health, they have inherent limitations, as some scales may not fully capture the heterogeneity and complexity of psychiatric conditions across clinical and cultural contexts [52]. Incomplete assessments of symptoms in this study may have limited statistical power and reduced the ability to more thoroughly explore sex-specific metabolic signatures. Future studies should incorporate larger, more diverse cohorts to enhance statistical robustness and better capture the influence of sex and other sociodemographic variables on metabolic profiles. In addition, future work would benefit from the integration of more thorough clinician-verified diagnoses. Because subgroup sizes were small and uneven once stratified by sex and distress category, formal statistical comparisons of sociodemographic variables between groups were not performed. Incorporating linear regression models that control for socioeconomic status, cultural background, and health disparities would also increase the generalizability and equity relevance of the findings.

Additionally, the cross-sectional design precludes longitudinal within-subject analyses, which are necessary to account for dynamic changes due to circadian rhythms, lifestyle, and nutrition. Several additional confounders may also have influenced the observed metabolic variation. Pre-existing health conditions (such as diabetes or hypertension), cultural or ethnic differences within the Syrian and Kurdish subgroups, and lifestyle-related factors, including medication use, sleep patterns, diet, caffeine consumption, smoking, and oral health, were not systematically controlled and may provide alternative explanations for some metabolic differences.

Finally, while saliva provides a practical, non-invasive sampling method, its composition reflects a filtered subset of blood metabolites, often at different concentrations [40,92]. Thus, complementary ^1^H NMR spectroscopy using blood samples may offer greater sensitivity and resolution in detecting subtle metabolic signatures associated with mental distress and resilience. It should be noted, however, that pathway enrichment results represent only a subset of pathway metabolites among the significant bins. These analyses serve to contextualize broad metabolic patterns rather than identify specific pathway perturbations. Although related salivary biomarker studies (e.g., amino acids, cortisol) suggest that saliva can reflect peripheral physiological responses to stress, these markers do not map directly onto disorder-specific neural mechanisms and should be interpreted cautiously. In addition, future studies would benefit from a complementary approach in which both NMR and mass spectrometry methods are utilized. Several of the metabolites in biological pathways are not present at sufficient concentrations in saliva to be detected by an NMR approach. This resulted in the utilization of a higher significance threshold in the pathway analysis in the present study. Employing both techniques would provide a method that could detect more of the metabolites in each pathway and allow for the selection of a more robust significance threshold.

In the future, integrating metabolomic data with psychometric measures, validating findings in independent cohorts, and considering the moderate sensitivity and specificity of some models will be essential for determining potential translational applications. Future studies would further benefit from longitudinal sampling to capture dynamic metabolic changes, the incorporation of multi-omics approaches (such as proteomics or epigenomics), and the application of advanced statistical or machine learning techniques that account for key covariates and improve predictive accuracy.

## 5. Conclusions

The present univariate and multivariate analyses revealed characteristic salivary metabolic “fingerprints” associated with psychological distress. Several significantly altered metabolites may serve as potential sex-specific salivary biomarkers for depression and anxiety. Pathways related to energy metabolism were most affected by mental distress, alongside notable changes in sphingolipid and taurine/hypotaurine metabolism. These findings suggest that ^1^H NMR spectroscopy-based metabolomics offers a promising, non-invasive approach for innovative, complementary mental health and distress screening using easily accessible saliva samples. The observed metabolic signatures may provide an objective tool to assist in the diagnosis of depression and anxiety in vulnerable populations, enabling rapid screening and the delivery of targeted, personalized interventions to those at highest risk.

## Figures and Tables

**Table 1:** Main sociodemographic variables among study participants. M: mean; SD: standard deviation.

Sociodemographic	Female		Male		Total	
	n	%	n	%	n	%
Marital Status	26		32		58	
Single	5	19.2%	8	25.0%	13	22.4%
Married	21	80.8%	24	75.0%	45	77.6%
Age	26		30		56	
18–24	8	30.8%	7	23.3%	15	26.8%
25–34	6	23.1%	6	20.0%	12	21.4%
35–44	8	30.8%	10	33.3%	18	32.1%
45–54	3	11.5%	6	20.0%	9	16.1%
>55	1	3.8%	1	3.3%	2	3.6%
Accommodation	24		27		51	
Collective accommodation centre	1	4.2%	1	3.7%	2	3.9%
Own apartment (alone or with family)	23	95.8%	25	92.6%	48	94.1%
Apartment together with other people	0	0.0%	1	3.7%	1	2.0%
Ethnicity	25		27		52	
Syrian	20	80.0%	23	85.2%	43	82.7%
Kurd	5	20.0%	4	14.8%	9	17.3%
Employed	26		32		58	
Yes	2	7.7%	14	43.8%	16	27.6%
No	24	92.3%	18	56.3%	42	72.4%
Education in Years	**M (SD)**	**Range**	**M (SD)**	**Range**	**M (SD)**	**Range**
*n* = 56	6.6 (5.0)	0–19	8.3 (4.2)	1–20	7.5 (4.6)	0–20

**Table 2:** Significant metabolic pathways that passed statistical testing (*p* < 0.1) in female and male comparisons. Hits/Total indicates the number of metabolites identified over the total number of metabolites found in the pathway. Raw *p*-values were taken from MetaboAnalyst 5.0. Impact represents the significance of the pathway to the overall results of the comparison studied.

Group	Pathway	Hits/Total	Raw *p*	Impact
Female Composite	Taurine and hypotaurine metabolism	1/8	0.025574	0.42857
	Sphingolipid metabolism	1/21	0.066014	0.0142
	Glycolysis/Gluconeogenesis	1/26	0.081205	0.00021
	Glycoxylate and dicarboxylate metabolism	1/32	0.099173	0
Male Composite	Glycolysis/Gluconeogenesis	2/26	0.000804	0.00021
	Sphingolipid metabolism	1/21	0.040123	0.0142
	Pyruvate metabolism	1/22	0.042006	0
	Glycerophospholipid metabolism	1/36	0.068115	0.02423
Female Depression	Terpenoid backbone biosynthesis	2/18	0.007851	0.25397
	Caffeine metabolism	1/10	0.074988	0
Male Depression	Glycolysis/Gluconeogenesis	2/26	0.003896	0.00021
	Amino sugar and nucleotide sugar metabolism	2/37	0.007832	0
	Fructose and mannose metabolism	1/20	0.075082	0
	Pyruvate metabolism	1/22	0.082324	0
	Pentose phosphate pathway	1/22	0.082324	0.11955
Female Anxiety	beta-Alanine metabolism	2/21	0.012469	0.05597
	Glycolysis/Gluconeogenesis	2/26	0.018845	0.00021
	Riboflavin metabolism	1/4	0.03316	0.5
	Amino sugar and nucleotide sugar metabolism	2/37	0.03666	0.05035

## Data Availability

The data presented in this study has not been uploaded to an online database accessible to the public due to privacy restrictions. However, the corresponding authors will make the data available upon reasonable request.

## Supplementary Materials

The following supporting information can be downloaded at: https://www.mdpi.com/article/10.3390/metabo16040216/s1, Table S1: *p*-values of salivary metabolites found to be significant in comparisons tested in either the Mann–Whitney U test, or Variable Importance Analysis based on random Variable Combination (VIAVC). Metabolites for which more than one NMR resonance peak was identified as significant are represented as metabolite.1, metabolite.2, … metabolite.n. † Indicates metabolites that were present in two or more similar sex comparisons while ‡ indicates metabolites that were present in both sexes and/or multiple comparisons.
