# Supplementary material for: Salivary Metabolomic Signatures Associated with Sex-Specific Psychological Distress in Syrian Refugees: A Proof-of-Principle Study
Source: Metabolites. 2026 Mar 25;16(4):216. doi: 10.3390/metabo16040216 (PMC13117666; doi:10.3390/metabo16040216)
Supplement: Supplementary file 1 [file metabolites-16-00216-s001.zip › Supplementary Table S1 - Metabolites - Saliva.pdf]

Supplementary Table S1

Group	Bin	Metabolite	Chemical Shift	% Diff	MW p-value	Best Subset p-value
Female Composite	1	Formate	8.46	-35.66	---	2.38E-26
	36	Unidentified†‡	5.85	-58.83	---	4.27E-72
	110	D-Tagalose	3.86	10.45	---	7.89E-47
	127	Glucose.3 and Lactulose†	3.73	-0.85	---	3.07E-42
	175	1,9-Dimethyluric Acid	3.30	-7.96	---	3.72E-15
	184	O-Phosphoethanolamine.3	3.22	-6.32	---	2.57E-23
	237	Taurine	2.21	20.35	4.81E-03	---
	251	N-Acetylneuraminic acid.1†	2.07	22.47	---	3.69E-21
	292	Citramalic acid.2†	1.37	19.01	---	1.38E-16
	320	2-Hydroxy-3-methylpentanoic acid †	0.95	20.20	5.38E-03	---
Male Composite	33	Unidentified	6.39	5.38	---	5.39E-21
	36	Unidentified†‡	5.85	38.16	---	5.24E-107
	41	L-Acetylcarnitine†‡	5.62	36.38	---	3.18E-49
	42	Unidentified	5.60	34.46	---	3.79E-27
	76	Unidentified	4.16	8.27	---	3.48E-98
	151	Glucose.5†	3.52	-11.07	4.47E-02	---
	182	O-Phosphoethanolamine.2	3.24	1.72	---	8.10E-15
	231	Unidentified	2.36	8.72	---	9.91E-25
	249	Unidentified	2.10	9.12	---	6.18E-151
	293	Lactate.1†	1.34	-14.42	4.04E-02	---
	294	Lactate.2†	1.33	-15.26	4.04E-02	---
Female Depression	6	Caffeine	7.90	-3.36	---	6.55E-14
	36	Unidentified†‡	5.85	-57.42	3.33E-02	3.16E-38
	127	Glucose.3 and Lactulose†	3.73	4.42	---	6.88E-19
	128	Unidentified	3.73	-0.54	---	3.62E-51
	185	O-Phosphoethanolamine.4	3.22	-5.51	---	5.26E-07
	204	Citramalic Acid.1	2.76	6.20	---	5.80E-16
	206	Sarcosine	2.74	13.13	---	4.65E-16
	207	Mevalonic Acid	2.73	7.58	---	5.41E-13
	208	2-Isopropylmalic Acid	2.71	-0.24	---	3.00E-23
	212	Citric Acid	2.66	5.58	---	1.85E-07
	262	N-Acetylneuraminic Acid.2	1.84	12.31	---	2.84E-11
	263	4-Guanidinobutanoate	1.83	8.33	---	6.61E-10
	288	Unidentified	1.43	6.57	---	2.75E-16
	292	Citramalic Acid.2†	1.37	15.09	---	2.98E-47
	320	2-Hydroxy-3-Methylpentanoic Acid †	0.95	17.34	4.86E-02	1.34E-05

Male Depression	24	Unidentified	7.16	18.54	4.32E-02	---
	28	Unidentified	6.95	25.86	3.02E-02	1.28E-20
	36	Unidentified†‡	5.85	64.03	4.76E-03	3.24E-267
	64	D-Galactose	4.61	16.60	4.71E-02	---
	75	6-Phosphogluconic Acid	4.20	8.61	---	7.42E-54
	108	Unidentified	3.88	8.50	---	7.58E-18
	151	Glucose.5†	3.52	-10.64	3.62E-02	---
	223	Methyl isobutyl Ketone.1	2.45	18.47	2.07E-02	---
	224	Methyl isobutyl Ketone.2	2.43	20.95	3.62E-02	---
	293	Lactate.1†	1.34	-17.02	4.32E-02	---
	294	Lactate.2†	1.33	-17.78	3.62E-02	---
	296	L-Fucose.1	1.26	-19.18	1.02E-02	3.48E-16
Female Anxiety	4	Carnosine	8.20	-10.65		1.12E-09
	5	Riboflavin.1	7.97	-27.23	4.20E-02	
	7	Unidentified	7.61	-33.00	3.72E-02	
	21	3-Methylphenylacetic Acid	7.25	-22.83		1.51E-13
	36	Unidentified†‡	5.85	-72.20	6.21E-03	3.07E-162
	38	Uracil	5.80	3.49		8.14E-06
	41	L-Acetylcarnitine†‡	5.62	-18.83		1.44E-11
	57	Riboflavin.2	5.12	-43.17	4.20E-02	3.31E-29
	61	Glucose.1	4.67	29.17	4.73E-02	
	102	O-Phosphoethanolamine.1	3.96	-3.56		2.83E-28
	103	Unidentified	3.95	-6.846		2.3011E-51
	119	Glucose.2	3.78	26.31	2.90E-2	
	121	N-Acetylgalactosamine	3.77	14.22	4.20E-2	
	129	Unidentified	3.71	6.208		1.17022E-27
	131	Ethanol	3.68	4.605		9.37216E-93
	149	Glucose.4	3.54	5.862		1.01264E-23
	160	Glucose.6	3.46	21.28		1.36736E-07
	162	N-Acetylglucosamine	3.44	22.24		6.10693E-12
	169	Unidentified	3.36	12.79		5.30963E-18
	232	Methionine Sulfoxide	2.35	2.514		3.65858E-17
	251	N-Acetylneuraminic Acid.1†	2.08	1.058		2.47752E-20
	271	5-Aminopentanoate.1	1.67	-12.096		3.33E-4
	273	Unidentified	1.67	0.433		2.20662E-12
	274	5-Aminopentanoate.2	1.66	-13.475		3.38807E-06
	278	5-Aminopentanoate.3	1.62	-9.3088		4.48E-2
	298	L-Fucose.2	1.21	-0.2542		1.26796E-06
	307	Unidentified	1.14	7.85		1.14511E-12
